# Pilot study of an intervention to increase cultural awareness in research mentoring: Implications for diversifying the scientific workforce

**DOI:** 10.1017/cts.2018.25

**Published:** 2018-08-08

**Authors:** Angela Byars-Winston, Veronica Y. Womack, Amanda R. Butz, Richard McGee, Sandra C. Quinn, Emily Utzerath, Carrie L. Saetermoe, Stephen B. Thomas

**Affiliations:** 1 Department of Medicine, Center for Women’s Health Research, University of Wisconsin – Madison, Madison, WI, USA; 2 Northwestern University Feinberg School of Medicine, Chicago, IL, USA; 3 Department of Kinesiology, University of Wisconsin – Madison, Madison, WI, USA; 4 Department of Family Science, Maryland Center for Health Equity, University of Maryland, College Park, MD, USA; 5 Institute for Clinical and Translational Research, University of Wisconsin – Madison, Madison, WI, USA; 6 Department of Psychology, California State University, Northridge, Los Angeles, CA, USA; 7 Health Services Administration, Maryland Center for Health Equity, University of Maryland, College Park, MD, USA

**Keywords:** Intervention, cultural awareness, research mentoring, evaluation, cultural diversity

## Abstract

**Introduction:**

Innovative evidence-based interventions are needed to equip research mentors with skills to address cultural diversity within research mentoring relationships. A pilot study assessed initial outcomes of a culturally tailored effort to create and disseminate a novel intervention titled Culturally Aware Mentoring (CAM) for research mentors.

**Intervention:**

Intervention development resulted in 4 products: a 6 hour CAM training curriculum, a facilitator guide, an online pretraining module, and metrics to evaluate the effectiveness of CAM training.

**Method:**

Participants were 64 research mentors from 3 US research-intensive universities. Quantitative pretraining and posttraining evaluation survey data were collected.

**Results:**

Participants found high value and satisfaction with the CAM training, reported gains in personal cultural awareness and cultural skills, and increased intentions and confidence to address cultural diversity in their mentoring.

**Conclusions:**

Study findings indicate that the CAM training holds promise to build research mentors’ capacity and confidence to engage directly with racial/ethnic topics in research mentoring relationships.

## Introduction

Evidence of racism and race-based prejudice and discrimination in the biomedical sciences and health professions would be easy to ignore, were they not so well documented [1, 2]. However, clinicians and scientists are reticent to acknowledge, and sometimes “color blind” to, the realities of race and history among their colleagues and trainees [3]. Despite some progress in the past several decades, a critical need remains for improvement in the training and experiences of individuals from historically underrepresented groups in the scientific workforce, including but not limited to Native Americans, African Americans, Hispanic/Latinos, and Hawaiians/Pacific Islanders [4]. The persistent racial disparities in professional attainments, including earned degrees and awards of federally funded R01 and other grants [5, 6], exposes the fact that race and ethnicity matter in biomedical and health science careers. Because it is only human to maintain the *status quo* [7], deliberate and proactive behaviors are required to counteract factors that contribute to the observed racial disparities in academic and career outcomes. One of those factors documented in the scientific literature is access to evidence-based mentorship [5], particularly mentorship that embraces and celebrates the cultural diversity within mentoring relationships.

There are increasing calls for evidence-based approaches to training [4] and other interventions to equip research mentors with skills and strategies to address cultural diversity and to not ignore the realities of racism in the biomedical and health sciences, particularly in mentored research experiences. In the helping professions including medicine, public health, counseling, and nursing, cultural competence has been proposed as a means to grow the capacity of providers to deliver culturally respectful care and to promote physical and mental health equity [8, 9]. Evidence supports the beneficial effects of cultural competence training on the attitudes and skills of health professionals [10]. Far less attention has been given to the likely equally important role of cultural “competence” in research mentoring. Good intentions and good will, although necessary, are not sufficient for tackling issues such as race, power, and privilege in mentoring relationships. As stated by Wear *et al*. [11], good intentions must be accompanied by the skills that can facilitate dialogue and address conflicts.

Unconscious bias trainings are proving to be critical catalysts in helping faculty to become self-reflective, and to recognize and address their personal biases in clinical and research sciences in academia [12, 13]. Although such trainings are important, they may be insufficient to provide a deeper understanding of how and why we are affected by race/ethnicity [1] and, more so, how to address and respond to racial/ethnic matters in the social interactions that occur in research mentoring relationships. Research mentoring relationships are the primary mechanisms for growing the next generation of scientists [4], and they are also the contexts in which cultural, social, and psychological factors that frustrate the engagement and persistence of emerging scientists from racial/ethnic groups historically underrepresented (HU) in the sciences occur, including feeling invisible, unvalued, incompetent, discriminated against, isolated, and marginalized [3, 14–17]. We assert that developing a deeper understanding of the ways in which race, racism, and privilege can contribute to the racial/ethnic disparities in academic and career outcomes should be an essential component of research mentor trainings and that such trainings need to provide mentors skills to navigate these dynamics. In this paper, we describe a cultural awareness intervention with skill-building components aimed at supporting research mentors’ confidence to engage in and respond to sensitive topics related to race/ethnicity as they mentor diverse scholars, particularly those from HU groups. The intervention is delivered via a national initiative discussed in the following paragraph.

In response to the need for evidence-based approaches to training and mentoring of individuals in biomedical research career pathways, the National Institutes of Health (NIH) launched the National Research Mentoring Network (NRMN; www.nrmnet.net) in 2014. NRMN is a nationwide consortium of biomedical professionals and institutions collaborating to provide all trainees across the biomedical, behavioral, clinical, and social sciences with evidence-based mentorship and professional development programming. The goal of this NIH flagship initiative is to enhance the diversity of the NIH-funded research workforce. NRMN’s program models emphasize the benefits and challenges of diversity, inclusivity, and culture within mentoring relationships, and more broadly the scientific workforce. Although critical training of emerging scientists occurs within research mentoring relationships, very little attention has been given to assisting research mentors who are predominantly White in developing the skills required to effectively mentor a more diverse population of women and scholars from HU racial/ethnic groups. Achieving mentoring effectiveness along these dimensions requires going far beyond traditional cultural competency to acquiring an understanding of how culture, race, ethnicity, and other social identities influence these often lifelong research mentoring relationships and actually practicing skills to respond to these factors. This paper reports on findings from a pilot study assessing the development process and initial outcomes of an intensive, multi-institution effort led by the NRMN Mentor Training Core to create and disseminate a novel intervention to teach Culturally Aware Mentoring (CAM) to research mentors.

## Intervention Design

### Process of CAM Curricular Development

The CAM team consisted of 8 scientists (6 women, 2 men; 5 White, 3 African American) from varying disciplines (biochemistry, community and public health, humanities, psychology), career stages (e.g., early career professionals, tenured professors, associate deans), and from 4 US universities. We had a range and decades of experiences designing, implementing, studying, and administratively coordinating professional development and training interventions for individuals in academia and in the private sector, including research mentors. We held teleconferences from December 2014 to December 2016 to conceptualize, develop, and test the CAM training. We referenced peer-reviewed research from the social sciences and education regarding theory and best practices on behavioral change and strategies for promoting cultural awareness. Four theoretical foundations were key in guiding our approach to the curriculum development and intervention design.

1. Multicultural and feminist theories, specifically the seminal scholarship by Sue *et al*. [18], Collins [19], and Anzaldúa [20]. These scholars asserted the importance of acknowledging all individuals as cultural beings (i.e., we all have culture, not just those from historically marginalized groups), the role of power and privilege in social interactions, and that individuals’ contexts must be considered in order to understand and intervene on their behavior.
2. Critical race theory as articulated by Solorzano and Yosso [21] based on their studies of Latino/a students’ persistence in higher education. This theory emphasizes the permanence of racism in US institutions and society, as well as the intersection between race and power. Solorzano and Yosso have investigated how the dominant culture undergirding predominantly White institutions can have an impact on the academic functioning and well-being on students from historically marginalized racial groups. Their work also documents the types of social capital such students have that allow them to be resilient in higher education, particularly at predominantly White institutions.
3. Transtheoretical model/motivation theory articulated by Prochaska and DiClemente [22]. Their seminal writings on smoking cessation articulated how behavioral change occurs across stages and identified several processes involved in behavioral change, including self-efficacy, decisional balance (i.e., weighing the pros and cons of new behaviors), and contingency planning for when new actions do not immediately result in desired outcomes.
4. Institutional transformation theories as captured in the National Science Foundation’s ADVANCE initiative and investigated by Fox [23, 24]. This body of work addresses several facilitating factors needed for systems change toward creating equity in access to resources and professional opportunities that improve achievement and advancement outcomes for women in academic science, technology, engineering, mathematics, and medicine (STEMM).

Informed by assertions in these theoretical perspectives and frameworks, we defined *culturally aware mentoring* as mentoring practices in which mentors recognize their own culturally shaped beliefs, perceptions, and judgments and are cognizant of cultural differences and similarities between themselves and their mentees. Such mentoring requires that mentors (1) gain intrapersonal cultural awareness, (2) interpersonal cultural awareness, and (3) skills to recognize and respond to cultural diversity issues that may arise in their mentoring relationships. These 3 factors constitute the 3 elements of the CAM training intervention.

### Stage 1: Training Conceptualization

We developed learning objectives, goals, and guiding principles for the training, building upon the CAM team’s assessment of the theoretical and evidence base from the scholarship cited above. We articulated 4 learning objectives: (1) identify how your cultural beliefs, worldviews, and identities influence your mentoring practices; (2) recognize how cultural diversity can affect—complicate *and* benefit—your research mentoring relationships; (3) acknowledge the impact of conscious and unconscious assumptions, privilege, stereotype threat, and biases on the mentor-mentee relationship; and (4) apply evidence-based strategies using case studies to reduce and counteract the impact of biases, stereotype threat, and privilege to foster trusting, culturally responsive mentoring relationships.

### Stage 2: Training Development

We developed activities aligned with the objectives and identified readings that could serve as reference material for participants. We solicited feedback on our training from a group of NRMN Master Facilitators, comprised of faculty and staff with advanced skill and experience in facilitating research mentor training. Their feedback guided our refinement of the CAM training content using an iterative cycle of creating or collecting, evaluating, revising, and finalizing key documents.

We based the CAM format on the research mentor training approach in *Entering Mentoring* [25, 26] that is well established and has been rigorously tested. The philosophy underlying *Entering Mentoring* emphasizes the development of mentoring principles, not specified mentoring practices, to guide participants discovering their own approaches for applying those principles to their practice. They do so by discussing common scenarios related to challenges in research mentoring relationships and then generating solutions to those challenges through group discussion. In the same vein, we approached development of CAM principles of practice based on research evidence to guide mentors in building their awareness of cultural diversity, especially racial/ethnic diversity awareness. Similar to *Entering Mentoring*, the CAM training rests upon a process-based approach, using case studies and group discussion about dynamics related to race and ethnicity to generate new insights related to CAM. We designed CAM training as a supplemental or advanced training for mentors who have participated in foundational research mentor training.

We used a face-to-face working meeting to (1) collectively review and discuss key multimedia material to incorporate into the training that could catalyze cultural awareness and rich discussion, (2) decide on sequencing of CAM curricular content, and (3) outline the CAM facilitation guide. The working meeting allowed us to experience the content and catalyzed several curricular decisions during and after the meeting. We decided to focus the CAM content specifically on race and ethnicity for 2 reasons. First, based on research, the hardest topic for most research mentors, especially White-identified mentors, to address is race/ethnicity, with some research mentors tending toward racial color-blind attitudes [27]. Second, we reasoned that if we can begin to address the challenges related to engaging with and addressing race/ethnicity in general and in our mentoring relationships in particular, then we can transfer those insights and learnings to addressing other aspects of cultural diversity such as those related to gender, socioeconomic status, mobility/ability status, and sexual orientation. We also decided to use a pretraining activity called the Culture Box to enhance participants’ understanding of their personal cultural identities and ready them to discuss these identities in small groups at the onset of the training. This activity instructs participants to prepare a “Culture Box” before the training that includes artifacts (actual or pictures of the artifact) that relate to any of their cultural identities. We did not limit their cultural identities to their race or ethnicity, but allowed them to determine the identities that were most important to them to share during the training; this sharing also can include how these shared identities can have an impact on their mentoring relationships. After the working meeting, continued team discussions prompted us to add content summarizing the psychological research explaining the science underlying concepts such as implicit bias and stereotype threat, as they may be unfamiliar to some research mentors.

The activities in Stage 2 resulted in a 6-hour training focused on enhancing both intrapersonal and interpersonal cultural awareness and cultural skill acquisition toward being an effective research mentor. The training is typically scheduled from 9:00 am to 4:00 pm (inclusive of a 1-h lunch break), is designed to be co-led by 2 facilitators, and consists of 3 sections. The “intrapersonal,” or self-reflection, section (2 h) provides an orientation to the training and includes introductory activities and exercises to engage participants in personal reflections about racial and ethnic identity. The “interpersonal” section (1 h) provides examples and research findings of how cultural diversity factors may operate in research mentoring relationships and implications for being a culturally aware mentor. Participants review key terms and view videos related to cultural diversity and learn more about the research behind bias and stereotyping. The “skill-building” section (3 h) illustrates racial/ethnic issues via case studies, outlines CAM principles, and uses role plays to provide participants an opportunity to apply and practice the principles. The 3 sections in the CAM training and example activities are highlighted in Table 1.

**Table 1:** Culturally Aware Mentoring (CAM) training areas of focus and example activities

Areas of focus within training and description	Example activities
*Part 1: intrapersonal/self-reflection (2 h):* participants are introduced to the structure of the training, ground rules are established, and the group engages in several activities designed to encourage mentors to explore their cultural identity	∙ “Culture Box.” Cultural identity activity (required homework) ∙ Racial identity exercise ∙ Self-reflection statements
*Part 2: interpersonal (1 h):* participants begin to explore how cultural identities affect interactions between mentors and mentees. Participants deepen their understanding of key terms and definitions introduced in the pre-session online module and discuss research on bias and stereotyping	∙ “A Tale of O: On Being Different.” Video on cultural diversity in organizations ∙ Key terms and definitions; exploring the science behind assumptions
*Part 3: skill-building (3 h):* Participants are introduced to strategies for culturally aware mentoring and are encouraged to build and practice skills through several case study and role-playing exercises	∙ Case studies ∙ Role play ∙ Principles and resources for culturally aware mentoring

### Stage 3: Taking the Show on the Road: Pilot Testing and Iterative Revisions to the Training

Pilot testing took place in 2016 at several universities. In addition, activities from the CAM training were offered as part of conferences and professional development interventions for faculty and staff involved in research training. All implementations of the CAM training were delivered by CAM team members in pairs of cofacilitators. We were purposeful in pairing cofacilitators who varied across demographics, including career stage, gender, disciplinary training, and racial/ethnic identities. We did this to provide participants with the opportunity to hear different perspectives and voices throughout the training, so that no facilitator felt like they had to be the authoritative voice on a particular topic. In this paper, we report on implementation of the full, 6-hour CAM training that occurred at 3 separate sites. The sites were affiliated with the NIH-funded Diversity Program Consortium, which includes the BUILD program (www.nigms.nih.gov/training/dpc/Pages/build.aspx) and NRMN. These initiatives have as their goal to diversify the research workforce by engaging and retaining trainees from diverse backgrounds in biomedical research, and by supporting diversity at student, faculty, and institutional levels through innovative approaches to research skill-building and training and mentorship. Each of the 3 sites initiated contact with the CAM team after learning about the training opportunity through NRMN and agreed to participate in the pilot testing of the CAM intervention. We took an iterative approach to our curricular design, informed by formative evaluation feedback collected through our surveys and conversations with training participants and facilitators across implementations. Next, we describe the implementations and subsequent changes that we made to the CAM curriculum based on participant and facilitator feedback.

Implementation 1 (n=14) occurred at a private historically Black university in a Southern US state (Winter 2016). A Black female social scientist employed as a research scientist and a White male basic scientist employed as a tenured professor and senior administrator served as the cofacilitators. Both facilitators had experience delivering professional development trainings to faculty and students, with active research programs investigating cultural diversity factors in the career development of HU racial/ethnic groups in the research sciences. On the basis of feedback from the participants and facilitators in Implementation 1, we added time for silent, self-reflection during brief moments throughout the training for participants to write any personal reactions, insights, or questions that emerged. We also incorporated brief descriptions of theoretical paradigms and concepts including White fragility [28] and systems-level thinking [24]. We implemented the revised training at 2 additional sites.

Implementation 2 (n=26) occurred at a large public university in a Western US state (Spring 2016). The same White male basic scientist from Implementation 1 along with a Black female social scientist served as the facilitators. Both were senior tenured professors. The social scientist was a trained therapist and researcher with extensive experience in designing and developing culturally relevant mentor training interventions, and an active research program—similar to the cofacilitator basic scientist—investigating academic and career development of HU racial/ethnic groups in the research sciences. Feedback from Implementation 2 included requests for additional time dedicated to skill-building, inclusion of more research findings and resources related to cultural diversity factors in research training, and definitions of cultural diversity terms (e.g., stereotype threat). As a result, we refined our case studies and devoted more time for participants to practice the CAM principles before the third implementation.

Before the third pilot, the CAM team decided to create a pretraining module delivered online for participants designed to be completed within a week before a scheduled training. This pretraining module off-loaded some of the time allotted during the training to cover foundational content, such as definitions of key terms and research on the relevance of race, ethnicity, and other dimensions of cultural diversity to research training. To create the online module, the CAM team curated extant articles and videos, and then created original narrative content to integrate the curated material into a coherent presentation. The goal of this online module is to serve as a primer for the training by increasing participants’ understanding of how cultural diversity issues are relevant to research trainees’ development, academic outcomes, and success. This online pretraining module addresses 4 topics: (1) race and privilege, (2) the experiences of scientists from historically underrepresented groups, (3) the realities of cultural diversity in the sciences, and (4) the role of CAM in trainee outcomes. Each section concludes with self-reflection questions and provides a “Go Deeper” set of relevant readings (e.g., New Yorker article, “The Origins of Privilege” [29]) and video clips (e.g., PBS series, “Race: The Power of an Illusion” [30]) for additional learning should participants choose. The online module is self-directed and takes about 1 hour to complete. This pretraining content allowed more time during the CAM training for participants to spend in skill-building. The online module was tested with 30 NRMN Master Facilitators from a range of disciplinary backgrounds and career stages who provided formative feedback on the module before its use with the final pilot-testing site.

Implementation 3 occurred at a graduate-serving institution in a Western US state (Fall 2016). The White male basic scientist from Implementations 1 and 2, the Black female social scientist from Implementation 2, and a White woman health scientist served as the facilitators. The health scientist was a senior tenured professor with extensive national leadership in developing and designing training programs for HU racial/ethnic groups and an active research program in health disparities. Participants (n=30) were part of a statewide advisory group on mentoring in academic STEMM departments and training programs and represented several colleges and universities. Formative evaluation from this pilot test resulted in additional minor edits and modifications to the curriculum (e.g., refining transitions between CAM sections, refining instructions for role plays in the case studies). Data for all 3 sites were examined to assess the value of this training and participants’ self-reported skill gains relative to CAM.

## Method

### Participants

A total of 70 mentors participated in the training across 3 implementations; 64 mentors (91%) provided consent for their data to be included in this research across the 3 pilot test implementations. Prior mentoring experience and demographic information for participants at each implementation site are provided in Table 2.

**Table 2:** Summary of demographic information and prior mentoring experiences of participants

	Implementation 1 (n*=*12)	Implementation 2 (n=26)	Implementation 3 (n=28)
Race/ethnicity of participants [n (%)]*			
American Indian/Alaskan Native	–	1 (4%)	–
Asian	1 (8%)	6 (26%)	2 (7%)
Black/African American	4 (31%)	1 (4%)	7 (25%)
Native Hawaiian/Pacific Islander	–	–	–
White	7 (54%)	13 (57%)	16 (57%)
Other	–	–	1 (4%)
Hispanic/Latino(a)	–	5 (22%)	4 (14%)
Not reported	–	1 (4.3%)	1 (4%)
Gender of participants [n (%)]*			
Male	4 (31%)	7 (30%)	7 (25%)
Female	8 (62%)	16 (70%)	20 (71%)
Not reported	1 (8%)	–	1 (4%)
Primary mentor to a student researcher [n (%)]	9 (75%)	22 (85%)	15 (52%)
Years of experience as a research mentor [mean (SD)]	14.22 (9.07)	11.55 (8.94)	14.64 (9.51)
Participated in prior mentor training [n (%)]	6 (50%)	19 (73%)	9 (31%)
Career stage of mentees [n (%)]*			
Junior faculty	1 (8%)	8 (31%)	15 (52%)
Postdoctoral fellows	1 (8%)	–	10 (35%)
K awardees	–	–	3 (10%)
T awardees	–	1 (4%)	3 (10%)
Clinical fellows	–	–	7 (24%)
Ph.D. or Master’s students	–	17 (65%)	15 (52%)
Medical/Healthcare Professional Students	1 (8%)	2 (8%)	11 (38%)
Undergraduates	9 (75%)	19 (73%)	10 (35%)
High school students	–	1 (4%)	1 (3%)

* Participants were invited to check as many categories as applied to them. As a result, column totals may add up to over 100%.

### Data Sources

Data were collected from mentors via surveys that were administered before and immediately after the training. Pretraining and posttraining data were collected via Qualtrics, an online survey administration tool. For this paper, we focus on data collected in the posttraining survey. Questions were selected from a library of metrics being used across NRMN [31] as part of their ongoing evaluation efforts. We augmented NRMN evaluation questions by including additional items that assessed the extent to which participants perceived gains in their cultural awareness and CAM skills.

#### Perceived Value of Training

We evaluated the value of the training to participants by assessing their likelihood to recommend the training to other mentors, by their ratings of the training facilitators, and by the perceived value of each activity implemented during each training. Specifically, mentors were asked “How likely are you to recommend this training to other mentors?” Response options ranged from 1 (v*ery unlikely*) to 5 (v*ery likely*). Mentors were asked to rate each facilitator as either *excellent, good, fair,* or *poor.* Finally, mentors in Implementations 2 and 3 were asked to rate how effective each topic or activity was in helping them to become a more culturally aware mentor; response options ranged from 1 (*very ineffective*) to 5 (*very effective*).

#### Perceived Gains in Skill

At the conclusion of the training, mentors retrospectively rated their level of skill before and after the training in several areas related to CAM. Four skill areas, which were assessed consistently across the 3 implementations presented in this paper, are reported in the results. Included in the survey was space for open-ended responses, in which participants provided additional comments about their experience.

### Analyses

All descriptive statistics and statistical tests of significance were calculated using IBM SPSS Statistics version 23. To examine *training satisfaction*, we calculated the percentage of mentors’ likelihood of recommending the CAM training to other mentors across each implementation. We then calculated mentors’ median rating of the training facilitators in each implementation. Next, we examined the average rating of the efficacy of activities in helping participants to become a more culturally aware mentor to determine the top 3 activities across each implementation.

Dependent samples *t-*tests were conducted for each of the 4 *skill gain* items to examine whether significant changes in perceived skill gains emerged. In addition to examining *p* values to determine statistical significance, we also examined practical significance using the effect size *d*_z_, which is a measure of the effect size of the standardized mean difference, 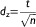 [15].

### Qualitative Interviews 18–24 Months Post Intervention

To begin understanding the long-term utility and influences of the CAM intervention, participants are being contacted 18–24 months after the training. A semi-structured interview protocol was used for phone interviews with willing participants. Interviews were conducted by 3 members of the CAM team who did NOT participate in the training at that site. All of those who did the interviews are highly experienced qualitative researchers. Phone interviews were audio-recorded and transcribed professionally for content analysis. For this pilot study, initial analyses are focused only on examples of changes in thinking and behavior, not attempting to relate to analytic framework or theory.

## Results

### Perceived Value of Training

The majority of participants were either likely or very likely to recommend the training to other mentors across Implementations 1 (n=11, 85%), 2 (n=23, 100%), and 3 (n=24, 85%) and rated the CAM facilitators highly (data not shown). As summarized in Table 3, the activities rated as most effective for helping mentors to become more culturally aware for Implementations 2 and 3 were “A Tale of O,” a video on cultural diversity in organizations, a case study and role play activity titled “Trainee Differences,” and the Culture Box. Our observations as facilitators were consistent with mentors’ high ratings of the Culture Box, as it was effective in getting mentors to open up quickly in sharing and reflecting on cultural diversity. This activity elicited strong emotions and authentic exchanges among participants, and it was not uncommon for individuals to be visibly moved or emotive while sharing their Culture Box content. It is the training activity that was the hardest to conclude, as mentors were highly engaged in respectfully displaying their cultural selves, many doing so for the first time. Two mentors from Implementation 2 noted that the Culture Box was “*helpful to break the ice and build an open conversation*” and “*allowed me to know my colleague’s story*.” Several noted the irony between how much they learned about colleagues in the room in 30 minutes through this activity in contrast to how little they know about their trainees they work with every week and for several years.

**Table 3:** Activities rated as most effective* for helping mentors become more culturally aware

	Implementation 2 [mean (SD)]	Implementation 3 [mean (SD)]
A Tale of O: video on cultural diversity in organizations	4.39 (0.58)	4.67 (0.56)
Culture Box: cultural identity activity	4.38 (0.50)	4.79 (0.42)
Case study and role-play activity: trainee differences	4.27 (0.77)	4.68 (0.73)
Case study: family ties	4.23 (0.61)	4.54 (0.51)
Principles for culturally aware mentoring	4.13 (0.62)	4.62 (0.57)
Research on cultural diversity dynamics	4.00 (0.76)	4.32 (0.72)
Discussion of *Nature* article, “Building a Future Scientist”	4.00 (0.69)	4.00 (0.85)
“The one thing you can do”	3.94 (0.57)	–^†^
Definition and discussion of key terms	3.86 (0.64)	3.81 (0.75)
Racial/ethnic identity exercise	3.83 (0.71)	4.57 (0.57)
Self-reflection exercises	3.63 (0.76)	–^†^

* Responses could range from 1 (*very ineffective*) to 5 (*very effective*).

† This activity was not included in Implementation 3.

Open-ended responses from participating mentors reflected the overall utility of the training. Some mentors commented on their own revelations regarding the importance of addressing cultural diversity in the research mentoring relationship. One mentor from Implementation 1 noted: “*This topic is important and worth the time it takes in meeting (e.g., building in time in meeting for discussion). It [culturally aware mentoring] is my ethical responsibility if I am going to be a mentor. Loved talking to my peers about this*!” Another mentor from Implementation 3 shared that “*I hadn’t thought about how these practices were important in inviting productivity in a lab*.” A mentor from Implementation 2 expressed appreciation for the research findings shared in the training: “*I will continue to advocate for my students and thanks to you I have research to support what I have [experienced]*.”

### Perceived Skill Gains

The average perceived skill level as retrospectively assessed by mentors in all 3 implementations is reported in Table 4. Significant skill gains were reported across all 4 skills as a result of attending the CAM training. The largest mean differences reported by mentors were detected for the skill “*Intentionally creating opportunities for my mentees to bring up issues of race/ethnicity when they arise*.” This large skill gain observed relative to intentions was mirrored in mentors’ responses to a question on how they intend to apply what they have learned in the CAM training. Many gave concrete examples of how they would mentor differently in the future. One example of such transformational plans to address issues of race/ethnicity came from a mentor who participated in Implementation 2: “*I’ll be more likely to bring up race/ethnic cultural issues as opposed to being open to them being discussed*.” This mentor’s intention reflects a shift from placing responsibility on the mentee to bring up discussions of race and ethnicity to being more intentional in initiating such discussions.

**Table 4:** Perceived culturally aware mentoring skill gains* as reported by mentors at the conclusion of the training

	Implementation 1 (n=13)					Implementation 2 (n=23)					Implementation 3 (n=27)				
	Before [mean (SD)]	Now [mean (SD)]	*M*_*diff*_ ^†^	*p*	*d*_*z*_	Before [mean (SD)]	Now [mean (SD)]	*M*_*diff*_ ^†^	*p*	*d*_*z*_	Before [mean (SD)]	Now [mean (SD)]	*M*_*diff*_ ^†^	*p*	*d*_*z*_
Intentionally creating opportunities for my mentees to bring up issues of race/ethnicity when they arise	4.38 (1.50)	5.62 (1.12)	1.23	0.004	1.00	3.70 (1.11)	5.26 (1.05)	1.57	<0.001	1.58	3.85 (1.51)	5.70 (0.82)	1.85	<0.001	1.50
Encouraging mentees to think about how the research relates to their own lived experience	5.00 (1.35)	5.77 (0.93)	0.77	0.006	0.92	4.39 (1.03)	5.52 (0.79)	1.13	<0.001	1.63	4.26 (1.48)	5.52 (1.22)	1.26	<0.001	1.15
Going outside of my comfort zone to help mentees feel included in the lab	4.62 (1.39)	5.62 (1.26)	1.00	0.006	0.93	4.35 (0.98)	5.70 (0.82)	1.35	<0.001	1.37	4.56 (1.56)	5.72 (1.17)	1.16	<0.001	1.29
Respectfully broaching the topic of race/ethnicity in my mentoring relationships	4.62 (1.71)	5.54 (1.27)	0.92	0.011	0.83	3.83 (1.03)	5.43 (0.84)	1.61	<0.001	1.92	4.19 (1.33)	5.63 (1.01)	1.44	<0.001	1.33

* Mentors were asked “Please rate how skilled you feel you were BEFORE the training and how skilled you feel you are NOW in each of the following items.” Responses could range from 1 (*not at all skilled*) to 7 (*extremely skilled*).

† *M*_diff_ represents the mean difference between mentors’ self-reported level of skill thinking back to before the training as opposed to now, after the training.

Importantly, both from observation and evaluation data, our approach to facilitating mentors’ critical self-reflection on who they are as cultural beings increases their understanding of the relevance of race and ethnicity in their research mentoring relationships. The findings also suggest that our training is effective in increasing participants’ perceived cultural skills. Finally, the day-long commitment (7 h inclusive of lunch break) did not appear to be a deterrent to participation and may be reflective of institutional commitment and faculty demand for more support. As one mentor from Implementation 2 wrote in the evaluation, “*This type of training is doable! (I doubted it before).*”

### Value of CAM Pretraining Online Module

In Implementation 3, 29 of the 30 participants (97%) completed the CAM pretraining online module. Most completed the module in 60–90 minutes (n=11) or 30–60 minutes (n=9), with a few spending either 90 minutes–2 hours (n=5) or more than 2 hours (n=4). Participants reported being familiar with the module topics before completing it [median=4 on a 5 point scale ranging from 1 (not at all familiar) to 5 (extremely familiar)], yet still rated the module components as highly valuable in preparing them to participate in the CAM training. On a 5-point scale (1=not valuable, 5=extremely valuable), the highest-rated module component was the videos (mean=4.73). One participant stated, “*I watched some videos 2xs, because so much info*.” Overall participant feedback and comments were favorable.

∙ Excellent—this was FASCINATING (original emphasis), educational, insightful, and really prepared (“primed”) us for discussion
∙ I feel the length was really ideal
∙ I learned the most from watching the entire hour-long piece “White like me.” I appreciated the historical perspective
∙ I liked that you could spend more or less time on each item
∙ More issues around gender difference
∙ Videos were very helpful, enjoyed having references available.

### Impacts of CAM on Participants Thinking and Actions After the Training

To determine the impacts or influences of the CAM intervention over time, an extensive interview-based qualitative study is underway. We invited participants to take part in an ~30-minute semi-structured interview with one of the CAM team members who did NOT lead the training at their site. Although this study is ongoing and will be the subject of future reports, some early insights into the types of impacts of CAM are emerging. Virtually all of the participants interviewed to date could easily identify some examples of lasting changes in their self-reflections and behaviors as a result of CAM. The themes identified from even these first analyses are summarized in Table 5.

**Table 5:** Impacts and influences of CAM from interviews 24 months after training

Greater realization of their own racial and ethnic biases and insensitivities
More comfort and proactivity talking with students about the importance of considering culture when engaging other people
Creating better communication within a research team—more listening of people’s different experiences definitely than before
Better engagement with historically underrepresented (HU) students, even by HU faculty
More awareness of how personal experiences vary and can influence behavior and performance, getting more information before jumping to conclusions
More awareness of how economic situations affect students
In one-to-one mentoring, checking in more on personal situations of students
Opening up to sharing more of himself so students can see how he is balancing work and life
More open-minded and seeking more information about how personal circumstances and factors can affect academic and research performance
Increased attention to help students problem-solve if they come from more difficult situations
More individualized mentoring strategies
More likely and confident to speak out when encountering false statement and biases related to experiences of diverse students
More comfortable in her own research that deals with racial/ethnic differences and health behaviors
More attuned to how choice of language in data interpretation and presentation in papers can be unintentionally negative toward specific groups
More comfortable having conversations with graduate students about language in writing

## Discussion

Colón Ramos and Quiñones-Hinojosa [3] asserted that although “we aspire to have a diverse biomedical workforce, conversations about why we lack diversity are frequently left to minority researchers.” Not only are conversations regarding persistent underrepresentation of specific racial/ethnic groups viewed by some as “not my problem or issue” [1], but conversations related to race, racism, and bias are viewed as irrelevant to research training and mentorship. These views are exacerbated by the fact that in the United States we are socialized to fear cultural diversity topics, especially those related to race and ethnicity [32]. It is no wonder that there are few places in academia where research mentors can have frank discussions about race, racism, and the legacy of these dynamics on our institutions and in the biomedical workforce [3]. The CAM training achieved the goal of initiating open, honest conversations about race, privilege, discrimination, unconscious bias, and the lived experiences of HU groups in the sciences. Moreover, the CAM training shows promise as an intervention to build research mentors’ capacity to engage directly with racial and ethnic topics in their research mentoring relationships.

Notably, mentors who participated in the CAM training reported significant skill gains not only in their intentionality to address race/ethnicity, but increased openness to broach racial, ethnic, and cultural topics in their research mentoring relationships and willingness to go outside of their comfort zone. Our evaluation data indicate that this increased openness to broaching was true even for faculty who were themselves from HU groups. The skills to enact culturally aware principles in research mentoring relationships are predicated upon the notion of racial stamina [28]. Racial stamina requires a willingness to mentally “hang in” through the discomfort often inherent in diversity dialogues, resisting the urge to divert, dismiss, or downplay race and ethnicity, and instead directly engage with these topics. Although DiAngelo [28] discussed racial stamina largely in the context of White majority individuals, who she identified as needing to increase their tolerance for racial stress in cross-racial dialogues, it is relevant across racial/ethnic groups. One reason that low racial stamina may occur is that individuals lack the strategies for navigating difficult dialogues. The increases that we observed in mentors’ perceived skill gains in enacting CAM principles suggest that the CAM training may support mentors’ racial stamina by providing them with evidence-based skills to facilitate addressing racial/ethnic dynamics in their mentoring relationships, including validating their trainees’ racial/ethnic and academic identities and discussing sensitive racial/ethnic topics.

In addition to the overall perception of training value expressed by mentors, the Culture Box activity was viewed as both useful within the context of the training and a tool that could be used in the context of their research mentoring relationships. Several mentors stated their intention to implement the Culture Box activity with their mentees or research groups. Others noted the utility of this activity as a tool for continued professional development with research mentors: “*I plan to take specific literature resources and activities such as the Cultural Box and videos and directly place them into the context of mentor development*” (mentor from Implementation 3). It was noteworthy how powerful the Culture Box activity was in quickly opening mentors up to exploring and sharing their personal cultural backgrounds with one another. As participants explore their personal cultural identities, it is impossible to anticipate the breadth and depth of content that they choose to share and their reactions to what is shared. Facilitators of CAM, therefore, must be especially nimble and alert to reading the emotional tenor of participants, gauging how to bring the activity to conclusion in a way that honors participants and what they disclose. Importantly, the Culture Box activity comes early in the CAM training just after ground rules are discussed, and sets the tone for the entire training thereafter, signaling to the participants an invitation to engage in authentic ways. The fact that this is the first activity is significant for 2 reasons.

First, what becomes quickly evident in the activity’s discussion is a pattern of which mentors’ bring artifacts that describe their racial/ethnic identity Versus other dimensions of cultural identity (e.g., gender, sexual orientation, physical mobility status, religious tradition). This observed pattern provides facilitators the opportunity to highlight the particular dynamics of being a member of a visible cultural group (e.g., racial/ethnic group) Versus a less visible cultural group (e.g., religious tradition). This pattern also highlights how we are socialized in the United States to view each other in terms of racial/ethnic group membership yet simultaneously avoid talking about race/ethnicity, and invites participants to consider the consequences of that avoidance in our everyday lives in general and in the research mentoring in particular. Second, the Culture Box activity emphasizes from the onset that we all have cultural identities. Mentors are subsequently encouraged throughout the remainder of the CAM training to consider cultural diversity, race/ethnicity in particular, in their mentoring relationships not from just their mentees’ vantage point but also from the vantage point of how their own cultural identities play out in the relationships. On several occasions, we were surprised to have observed participants self-disclose physical disability status, unresolved traumatic experiences, and racial/ethnic backgrounds not evident from phenotypic appearance with other members of their small group. These reflections were shared as potential sources of vulnerability that helped participants gain a degree of empathy with the lived experience of HU racial/ethnic groups. For these reasons, we chose to keep the instructions general for the Culture Box.

A sizeable percentage of participants, but not all, in the CAM pilot study had prior research mentor training. Although the CAM training may be useful to research mentors regardless of prior training, our experience with CAM training indicates that those with foundational knowledge of research mentoring principles may be better prepared to incorporate CAM content into their mentoring practices.

Finally, given that the training is nearly a full workday in length, one might expect that participants would experience exhaustion cognitively, emotionally, and physically. We did not specifically assess participant engagement or energy levels throughout the training. On the basis of facilitators’ observations, participants were experientially saturated at the conclusion of the training. Some participants reported on their evaluations that the training could be shorter (e.g., “*Doesn’t need to be 6 h long*”). Others reported to facilitators and on their evaluations that they would have welcomed more time, perhaps spread across 1.5 days with time in between to process new insights (e.g., “*More time to reflect before discussion and more case studies; allow more time for introspection and journaling”; “More time for discussion [is there ever enough time*?]”).

### Future Directions

Next steps for the CAM team include continued evaluation of the impact of the CAM training on participants. This evaluation will include follow-up (6 months, 1–2 years later) with the implementation sites in the pilot-testing phase to investigate how participation in the CAM training affects the longer-term attitudes, beliefs, and actual behaviors of mentors. Indeed, one significant measure of success will be the persistence of CAM training effects on both mentors’ behavioral changes and the academic and career outcomes of the trainees they mentor. Extensive research based on the theory of planned behavior, widely tested in health behavior models, demonstrates that intentions are the most important determinants of people’s eventual behavior [33]. A recent systematic review investigating empirical studies predicting self-care intentions and behaviors in individuals at risk of diabetes revealed that intention was the most predictive construct of self-care behaviors [34]. As reported in the Results section, our initial findings from follow-up interviews indicate that participants are subsequently making changes in their mentoring behaviors and related beliefs. Further evaluation will also allow us to examine what aspects of the CAM training mentors attribute to their skill gains.

In addition, the CAM team has recently trained more expert facilitators to lead CAM trainings, and they will be leading them in universities around the United States over the next 12–24 months. Although the basic design of the CAM session will remain constant, the pilot testing has revealed a need to adapt it slightly to the unique context of each site. Those adaptations will be documented, as will systematic feedback and observations of the cofacilitators after each training. Thus, we will continue to study not only the short-term and long-term influences of CAM on participants but also how it is best delivered as an intervention in a variety of different academic contexts.

### Limitations

The CAM training currently focuses on the demographic diversity variables of race/ethnicity. Although a significant number of participants self-identified as White, some mentors were from HU racial/ethnic groups. We also note that a majority of participants in our samples were women. Participants’ gender intersecting with race and ethnicity might be an important interactional effect to be investigated and addressed in future CAM trainings, including the effectiveness of the race/ethnicity-focused CAM training with greater numbers of male participants from racially/ethnically diverse backgrounds. The self-report nature of our evaluation data carries the limitations commonly identified with these data, including the question of to what extent participants’ behavioral intentions to practice culturally aware principles in their mentoring translate to actual behaviors. Thus, we caution the extrapolation of our findings based on our pilot test results and hope that these initial findings spark continued research on interventions to prepare mentors to be more effective with trainees who are different from themselves in any cultural dimension, toward the larger goal of advancing scientific workforce diversity.

### Conclusion

The CAM training is a novel culturally tailored curricular intervention for research mentors and has great promise to go beyond the surface and open deep self-reflective dialogue about race/ethnicity in science, research training, and academic medicine for which NIH leaders have been calling. Further, whereas cultural diversity trainings often raise participants’ awareness of personal and interpersonal cultural factors, the CAM training goes a step further and provides mentors with the opportunity to practice enacting CAM-related skills. The combined efforts of our CAM team have resulted in 4 products: a 6-hour training curriculum, a facilitator guide, an online pretraining module, and metrics to evaluate the efficacy of this training. Buoyed by the NRMN, we intend to make the training more broadly available and have begun training more facilitators to lead its continued implementation nationwide. The training is complex to lead as it rapidly opens up challenging conversations that facilitators must be prepared to guide, requiring solid skills in group dynamics and an ability to navigate cultural diversity factors that subtly and overtly emerge from and between participants. The training is not sufficient to change the face of science and research by itself. However, our evaluation data from this pilot study indicate that the CAM training is able to facilitate research mentors’ awareness of, intention to, and confidence in attending to racial and ethnic matters that must be addressed as we work toward equity and inclusion in diversifying science.
